# Respiration and metabolism of the resting European paper wasp (*Polistes dominulus*)

**DOI:** 10.1007/s00360-015-0915-7

**Published:** 2015-07-02

**Authors:** Helmut Käfer, Helmut Kovac, Barbara Oswald, Anton Stabentheiner

**Affiliations:** Institut für Zoologie, Karl-Franzens-Universität Graz, Universitätsplatz 2, 8010 Graz, Austria

**Keywords:** *Polistes*, Resting metabolism, Respiration, Adaptation, Environment, Insects

## Abstract

The European paper wasp, *Polistes dominulus* Christ, is an abundant wasp species in South and Central Europe which dispersed to the north in recent times. *Polistes dominulus* exhibits an energy-extensive mode of life, spending much time resting at the nest, which should be reflected in adaptations regarding gas exchange and standard metabolism. We analysed the resting metabolism (CO_2_ emission) of *Polistes dominulus* workers in the ambient temperature range an individual may be exposed to during a breeding season (*T*_a_ = 2.4–40.6 °C) via flow through respirometry. Behaviour and endothermic activity were assessed by infrared thermography. With rising *T*_a_, CO_2_ release followed an exponential increase from 27 to 149 and 802 nl g^−1^ min^−1^ at *T*_a_ = 3, 20 and 35 °C, respectively. Measurements of the thermal regime at the nest showed that resting *P. dominulus* are most of the time in the lower range of their standard metabolic curve. A comparison with a “highly energetic” wasp like *Vespula* sp. revealed that *Polistes dominulus* not only optimises behaviour but also reduces metabolism to save energy. The CO_2_ emission patterns changed with ambient temperature, from discontinuous (≤25 °C) to cyclic (25–36 °C) and continuous gas exchange at higher temperatures. A pronounced break appeared in the data progression regarding cycle frequency and CO_2_ emission per gas exchange cycle between 15 and 10 °C. This striking change in gas exchange features indicates a physiological adaptation to special respiratory requirements at low temperatures.

**Electronic supplementary material:**

The online version of this article (doi:10.1007/s00360-015-0915-7) contains supplementary material, which is available to authorised users.

## Introduction

*Polistes dominulus* is the most common paper wasp in Europe. *Polistes* builds small nests and the colonies have up to ~30 individuals. Brood is reared in an open, single-combed paper nest made of chewed wood and plant fibres. In warmer areas the nests are suspended on plants or rocks. In cooler areas, the nests are often found in sheds or attics, where they are attached to beams or roof tiles. *Polistes dominulus* is considered primitive eusocial due to the low caste dimorphism (Wilson 1971). Brood rearing is characterised by overlapping generations, cooperative brood care and reproductive division of labour. The species was originally native to South and Central Europe and Northern Africa. From there it has distributed to the east as far as China (Cervo et al. 2000). In the last 30 years the range of *P. dominulus* widened strongly to the north of Europe (Pekkarinen 1999, Smit 2003, Woydak 2006). It was accidentally introduced into North America (Eickwort 1978, Hathaway 1981), where it has spread throughout most of the country, competing successfully against the native paper wasp species (e.g. *Polistes fuscatus*) and replacing them (Cervo et al. 2000, Stahlhut et al. 2006). The distribution range and propagation of *P. dominulus* has drawn attention to the species’ extraordinary adaptability. The energetic performance is a crucial determinant for distribution and survival in a new and variable environment.

One could assume that *Polistes dominulus* has similar thermoregulatory capabilities as other wasps of similar size and body mass, e.g. *Vespula vulgaris* or *V. germanica* (Coelho and Ross 1996, Heinrich 1984, Kovac et al. 2009, Kovac and Stabentheiner 2012), but this is not the case. Though they can actively heat up their thorax, *Polistes dominulus* has significantly lower endothermic capability than *Vespula* sp., despite their similarity in size and weight (Kovac et al. 2009, Weiner et al. 2010). Insect metabolism is—amongst other factors—dependent on activity and body temperature, which are in turn heavily influenced by ambient temperature (Bartholomew and Lighton 1986, Chappell and Morgan 1987, Heinrich 1974, 1987). Standard metabolic rate (SMR) or resting metabolic rate (RMR) are measures of the basal energy performance of an ectothermic organism at a defined temperature (DeVries et al. 2013, Lighton and Fielden 1995, Moyes and Schulte 2008, Vogt and Appel 1999, Willmer et al. 2004). Assessment of the energetic costs for activity such as thermoregulation, flight or food procurement is only possible by comparison with the resting values. The aerobic capacity hypothesis postulates that an efficiency optimisation towards a high active metabolism results in a coincidentally high resting metabolism (Bennett and Ruben 1979, Pough and Andrews 1984). Flying insects tend to have a higher resting metabolism than wingless insects (Addo-Bediako et al. 2002), though contrary results were found in a comparison of winged and wingless ants (Lighton and Berrigan 1995). These considerations, however, focus not only on flight, but on energetically costly behaviour in general (Reinhold 1999). Insects with a less active way of life have a low resting metabolic rate (e.g. the ant lion (Lucas 1985) or the wood tick (Lighton and Fielden 1995)). *Polistes dominulus* also exhibits an energetically extensive mode of life. Their active metabolism (Weiner et al. 2012) is rather low in comparison to *Vespula* (Käfer et al. 2012) and honeybees (Harrison et al. 1996, Harrison and Roberts 2000, Stabentheiner and Kovac 2014, Woods et al. 2005). Individuals also spend much time sitting on the nest and often rest whilst foraging. They do not show endothermic thermoregulation even in cool nights—probably because the produced heat would be immediately lost to the environment (our own unpublished data; Höcherl and Tautz 2013, 2015). Therefore, it is important to know the impact of factors such as environmental conditions and way of life on energetic costs. In *P. dominulus*, however, resting metabolism is known at only one ambient temperature (25 °C; Weiner et al. 2009). In the present investigation, we measured their resting metabolism (CO_2_ emission) in the whole ambient temperature range an individual may likely be exposed to during a breeding season.

The external gas exchange of resting insects in many cases occurs via rhythmic spiracle activity and abdominal pumping movements. They show discontinuous gas exchange patterns (Hadley 1994, Hetz and Bradley 2005, Levy and Schneiderman 1966, Lighton 1996, Sláma 1999). These patterns change with temperature. Whilst in honeybees spiracle control fails already below about 10 °C (Lighton and Lovegrove 1990, Kovac et al. 2007), vespine wasps are still able to maintain spiracular function down to *T*_a_ = 2.8 °C (Käfer et al. 2013). During own observations we noticed that *Polistes* reduces voluntary activity at low ambient temperatures much more than vespine wasps with more pronounced endothermic capability (Kovac and Stabentheiner 2012). In order to see how respiratory gas exchange changes in dependence on activity at low temperatures we analysed these patterns in the whole range of ambient temperature occurring on a nest during the breeding season.

## Materials and methods

### Animals

The respiration experiments were conducted in summer 2011 (July, August) and 2013 (July). 35 *Polistes dominulus* Christ females were captured in an orchard, near the laboratory in Gschwendt, Styria, either when foraging or directly from nests situated under the roofs of nearby buildings.

The resting metabolism was investigated in a temperature range between 2.4 and 40.6 °C in steps of ~5 °C. Nine temperature categories were defined (2.9, 6.9, 10.3, 15.4, 22.4, 26.4, 31.0, 35.6, 40.3 °C; see also supplementary material, Table S1). In each temperature category we tested three to five individuals. Five individuals were exposed to two ambient temperatures (see Table S1). These individuals did not deviate from the 30 wasps tested at only one experimental temperature regarding the assessed activity, respiration and metabolism data. Therefore, all individual data were pooled. Most experiments (86.1 %) were executed overnight to increase the likelihood of the tested individuals being at rest over a maximum timespan. Because of the long experiment duration of 3–10 h the individuals were provided with 1.5 M sucrose solution ad libitum within the measurement chamber.

**Fig. 1:**
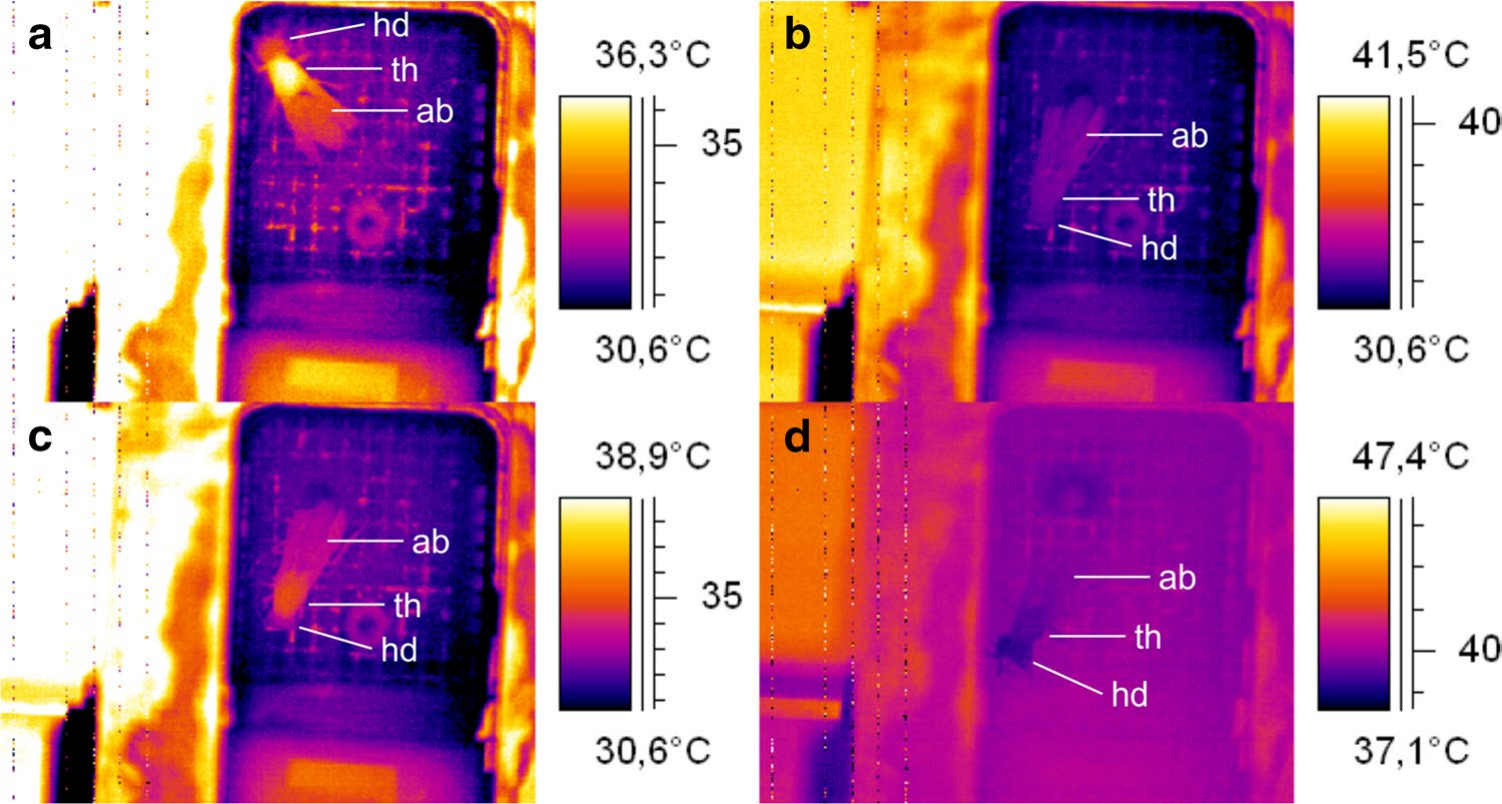
Thermograms of *Polistes dominulus* in different thermic states. Head (hd), thorax (th), abdomen (ab) are marked. **a** active, endothermic individual; **b** individual at rest, ectothermic, with all body parts at the same temperature; **c** resting individual, weakly endothermic (slightly elevated *T*_th_ over *T*_ab_); **d** resting wasp at high *T*_a_ = 40.3 °C: active evaporative cooling of head and thorax via regurgitated liquid

The individuals were weighted with a balance to the nearest 0.1 mg (AB104, METTLER-TOLEDO, Greifensee, Switzerland) before and after the experiment. They were put into the respiration measurement chamber immediately after being captured and weighted. The handling led to increased activity of the animals, which was reflected in walking or running, body movement or endothermy (Fig. 1a) and a high metabolic rate. Therefore, we started the measurements only at least 30 min after insertion to let the wasps calm down sufficiently.

To investigate the temperatures the insects may likely be exposed to during a breeding season, we measured the ambient temperature from April to October in 15-min intervals 1 cm above two nests (one in 2012 and one in 2013). Lower thermal limit experiments were conducted in September 2014 with nine individuals captured on two nests.

### Respiration measurement

We determined gas exchange via CO_2_ release by means of a flow through respiratory measurement setup (Kovac et al. 2007, Käfer et al. 2012, Stabentheiner et al. 2012). The wasps were put in a measurement chamber of 18 ml volume (3 × 3 × 2 cm) which was not hindering the individuals’ movement. The chamber was placed in an electronically controlled water bath (JULABO F33, JULABO Labortechnik GmbH, Seelbach, Germany) which kept the adjusted experimental ambient temperature (*T*_a_) within narrow margins (±0.3 °C). A thermocouple connected to a data logger (ALMEMO 2690, Ahlborn GmbH, Holzkirchen, Germany) recorded the temperature the animal was exposed to inside the measurement chamber (within 1 cm) in intervals of 1 s. Air was pumped through mass flow controllers (Brooks 5850S; 0–1000 ml/min; Brooks Instrument, Hatfield, USA) at a rate of 144 ml min^−1^. The outside air CO_2_ concentration was measured in the reference channel of a differential infrared gas analyser (DIRGA; Advance Optima URAS 14, ABB Analytical, Frankfurt, Germany) with ~2 ppm accuracy (sensitivity < 0.5 ppm), and then led through the measurement chamber and the DIRGA’s measurement channel. Data were recorded at intervals of 1 s. The DIRGA was calibrated at the start and at the end of the experiment and at 3 h intervals to zero and end point via internal cuvettes to correct possible offset and drift. To simulate natural conditions, relative humidity in the measurement chamber was maintained with a set of humidifying bottles, filled with distilled water, which were placed in a second JULABO water bath. The temperature in this water bath was regulated corresponding to that in the measurement chamber to keep the relative humidity constant (100 % at 2.5 °C, 81 % at 6 °C, 62 % at 10 °C, 50 % from 15 °C upwards; see supplementary material Fig. S2; see also Stabentheiner et al. 2012).

A gas exchange cycle was defined from the beginning of one open phase (i.e. CO_2_ concentration minimum before the burst started) to the beginning of the next open phase. The CO_2_ production rate (VCO_2_) was calculated by integration of 10 (or 5, see Sect. “Activity and body temperature”) minute intervals. For these intervals, the mean CO_2_ production rate was calculated. The number of evaluated time intervals per tested individual at a certain *T*_a_ varied up to sevenfold because of differences in the wasps’ behaviour. To prevent overrepresentation of single individuals, mean values of every individual were used to calculate the mean VCO_2_ per temperature. The CO_2_ emission per gas exchange cycle, cycle duration (min) and discontinuous gas exchange cycle phase durations were calculated from the single cycles in the evaluated intervals. The number of cycles available for evaluation at a certain *T*_a_ varied up to 15-fold between the individuals, e.g. because some individuals were much less active than others, which resulted in longer timespans of rest and therefore more evaluable intervals. To prevent overrepresentation of such individuals, mean values of the individuals in a temperature range were used for the calculations. This applies to all analyses regarding cycle and phase duration, cycle frequency, mean CO_2_ emission and CO_2_ emission per cycle at a certain *T*_a_.

Data analysis and statistics were done in Excel (Microsoft Corporation, Redmond, USA) with proprietary peak finding formulas, Origin Pro 8.1 (OriginLab Corporation, Northampton, USA) and Stathgraphics Centurion XVI (StatPoint Technology Inc., Warrenton, USA). The amount of CO_2_ release (nl g^−1^ min^−1^) reported in this paper refers to standard (STPS) conditions (0 °C, 101.32 kPa = 760 Torr).

### Activity and body temperature

**Fig. 2:**
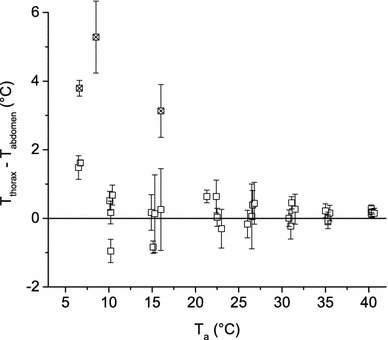
Thoracic temperature excess over the abdomen (*T*_thorax_ − *T*_abdomen_) in dependence on ambient temperature (*T*_a_).* Data points* represent individuals with mean values and SD (*N* = 3–5 individuals per temperature range, *n* = 52–252 thermograms per individual). Resting *Polistes dominulus* were mostly ectothermic or weakly endothermic. Exceptionally high values result from the animals’ position in the measurement chamber (marked data). High variance in individual values indicates alternating ectothermic and endothermic periods

To determine the wasps’ activity and thermic state during respirometric measurements we applied infrared (IR) thermography. The lid of the measurement chamber was covered by a plastic film transparent in the infrared range from 3 to 13 µm, which allowed us to observe the wasps and record their behaviour and body surface temperature (ThermaCam SC2000 NTS thermography camera, FLIR Systems Inc., Wilsonville, USA). Evaluation of the surface temperatures of head (*T*_hd_), thorax (*T*_th_) and abdomen (*T*_ab_) was done with AGEMA Research software (FLIR Systems Inc.) controlled by a proprietary Excel (Microsoft Corporation, Redmond, USA) VBA macro. Infrared data were stored digitally on a hard disc at a rate of 5–10 frames per second. The measured body surface temperature was calibrated to 0.7 °C accuracy, assuming a wasp cuticle infrared emissivity of 0.97 (Kovac and Stabentheiner 1999) and using a proprietary Peltier-driven reference source of known temperature and emissivity (for details see Schmaranzer and Stabentheiner 1988, Stabentheiner and Schmaranzer 1987, Stabentheiner et al. 2012). The infrared video sequences allowed quantification of even slight endothermy of the wasps (small thoracic temperature excess over the abdomen, *T*_th_ − *T*_ab_) over a longer resting period. In the lowest temperature range (*T*_a_ = 2.4–3.0 °C) we had to insulate the lid of the measurement chamber with Styrofoam, making IR recordings impossible. Evaluation of the corresponding CO_2_ recordings ensured that the tested individuals were at rest. “Rest” was defined as follows: no or only small visible body activity (i.e. only movements of antennae or single legs allowed) and ectothermic state (according to Crailsheim et al. 1999, Kovac et al. 2007, Stabentheiner and Crailsheim 1999, Stabentheiner et al. 2003) for at least 10 min. However, this condition was not always practicable. At high experimental temperatures (*T*_a_ > 30 °C) we had to evaluate 5 min intervals as well, as only few or no 10 min periods of continuous rest occurred. Because of the “window” in the measurement chamber’s lid a temperature gradient occurred at low temperatures. Some individuals positioned themselves in such a way in the gradient that their thorax temperature seemed elevated (Fig. 2).

It was not possible to evaluate respiration patterns in two individuals and CO_2_ per respiration cycle data in one individual. Two wasps did not show rest at all and had to be excluded from our analysis. Therefore, the number of analysed individuals (*N*) deviated from the number of tested individuals given in Sect. “Animals” (*N*_test_ = 35).

### Lower thermal limit measurement

Nine *Polistes* individuals captured on two nests in September 2014 were inserted into plastic vials of 14 ml volume (14 mm diameter x 100 mm length) each. A tenth vial was equipped with a thermocouple connected to an ALMEMO data logger which recorded the temperature the animals were exposed to during the experiment. The 10 vials were mounted in a self-constructed shaking device and submerged into a JULABO water bath described in Sect. “Respiration measurement”. After ~5 min of habituation we drove a temperature ramp from 15 °C to −5 °C with a dT of 0.25 °C min^−1^, maintaining the minimum temperature for 5 min and then increasing *T*_a_ to 15 °C within 10 min (dT of ~2 °C min^−1^). The individuals inside the chambers were forcefully shaken for 1 s in 1 min intervals. The experiment was recorded on video for later evaluation (Sony HDR-CX730E, Sony Europe Limited, Wien, Austria). The time (and therefore temperature) when coordinated righting movements ceased after the shaking was determined via behavioural observation.

## Results

### Weight

Body mass changed considerably in single wasps. The weight of the 35 individuals as determined at the beginning and at the end of each trial was 89.8 ± 15.4 mg and 90.7 ± 17.3 mg, respectively. Wasps did not lose weight during experiments on average; the mean weight gain in surviving individuals was 5.9 ± 12.8 mg (compare Fig. S2) with a maximum weight gain of 32.0 mg and a maximum weight loss of 15.4 mg. Individuals which died during the experiments were always lighter at the end with a mean weight loss of 29.4 ± 32.4 mg and a maximum loss of 73.3 mg.

### Activity and body temperature

At lower ambient temperatures (*T*_a_ < 20 °C) the wasps calmed down within a few minutes after being inserted into the measurement chamber and stayed at rest for long periods. At temperatures ≤10.3 °C the wasps were almost exclusively at rest for the entire experiment. The thoracic temperature excess over the abdomen (*T*_th_ − *T*_ab_) ranged from +5.3 °C to −0.9 °C (Fig. 2). Four of eight individuals were ectothermic (Fig. 1b) or only slightly endothermic (*T*_th_ − *T*_ab_ < 0.1 °C, Fig. 1c). At a *T*_a_ between 10 and 30 °C the wasps were quiescent most of the time, showing only weak endothermy with a thoracic temperature excess <1.0 °C. At high ambient temperatures (*T*_a_ ≥ 35.6 °C) they showed activity for most of the experiments’ duration, interrupted only by brief periods of rest and ectothermy. At these high temperatures, the wasps’ *T*_th_ − *T*_ab_ during rest was minimal (± 0.5 °C, Fig. 2). Evaluation of the IR video data revealed that at 26.4 °C two of five (40 %), at 31 °C one of four (25 %), at 35.6 °C three of five (60 %) and at 40.3 °C all four (100 %) individuals started to show evaporative cooling behaviour (regurgitated droplets of liquid to cool the head via evaporation, Fig. 1d).

A high standard deviation of the thoracic temperature excess (with a maximum of 1.19 °C) indicates alternating states of ectothermy and endothermy throughout an experiment (Fig. 2). High as well as low (negative) values in thoracic temperature excess at low experimental temperatures might partly have been caused by a temperature gradient inside the measurement chamber and the position of the tested individual in this gradient, although they were sitting still over the whole duration of the experiment, especially in three individuals at *T*_a_ = 5–16 °C, (Fig. 2, marked data points).

### Gas exchange patterns

**Fig. 3:**
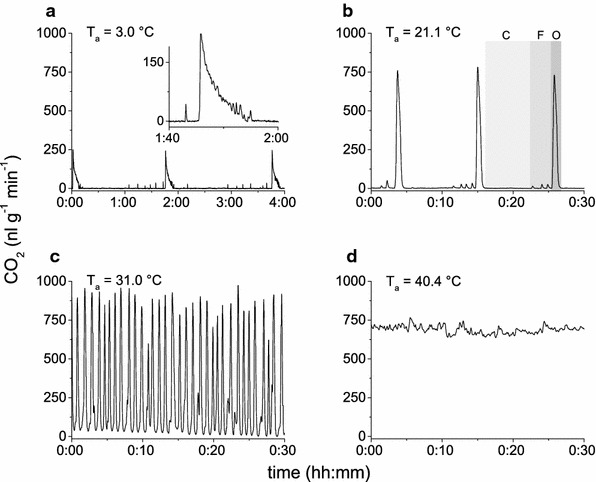
Representative gas exchange patterns of resting *Polistes dominulus* at different ambient temperatures (*T*_a_). CO_2_ emission changed in pattern and frequency with rising *T*_a_. **a** *T*_a_ = 3.0 °C; a flutter phase is visible before the main (open phase) CO_2_ burst. Bursts consist of one higher and subsequent lower, merging pulses (*insert*). **b** *T*_a_ = 21.1 °C; typical discontinuous gas exchange pattern with closed (*C*), flutter (*F*) and open (*O*, peak) phases. **c** *T*_a_ = 31.0 °C; cyclic respiration with residuals of flutter phases before some peaks. CO_2_ signal does not always reach zero. **d** *T*_a_ = 40.4 °C; continuous respiration

The CO_2_ concentration data were evaluated to obtain gas exchange patterns and CO_2_ production of resting individuals in dependence on ambient temperature. *Polistes* wasps released carbon dioxide discontinuously at *T*_a_ ≤ 26.4 °C (Fig. 3a, b) with distinct closed, flutter and open phases. At low experimental temperatures (*T*_a_ = 2.4 to 10.3 °C) the large (open phase) bursts consisted of an initial larger peak followed by subsequent, merging smaller ones (Fig. 3a), indicating spiracular activity. Between these bursts, smaller flutter events were recorded in a sometimes irregular pattern which not always allowed the definition of a definite closed phase. At higher temperatures, respiration followed the “classic” discontinuous gas exchange pattern (see e.g. Levy and Schneiderman 1966, Hetz and Bradley 2005, Lighton 1996) with closed, flutter and open phases (Fig. 3b). From 26.4 up to 35.6 °C, a cyclic gas exchange pattern was observed (Fig. 3c), with no detectable closed phases. Whilst the CO_2_ trace at *T*_a_ = 31.0 °C sometimes still showed residuals of flutter phases (compare Käfer et al. 2013), usually the CO_2_ concentration in the measurement chamber did not reach zero at this and higher temperatures (Fig. 3d). At *T*_a_ = 40.3 °C, periods of rest were rare and—if present at all—short. Therefore, data at this experimental temperature were sparse and, although included in the subsequent evaluation if possible, should be treated with caution. Gas exchange followed a continuous pattern, which did not allow depicting single peaks. Peaks merged, building rounded “hills” and “plateaus”.

### Cycle duration–phase duration–respiration frequency

**Fig. 4:**
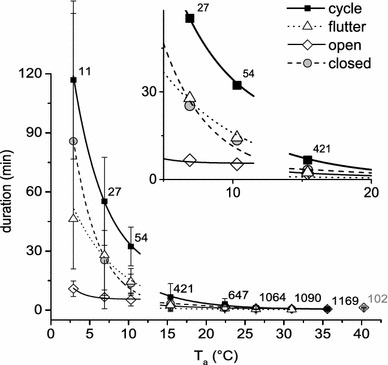
Duration of gas exchange cycles and cycle phases (*open*, *closed*, *flutter*; where present) in dependence on *T*_a_. Note interruption of data courses due to a striking “step” between 10 and 15 °C. Duration decline in all phase types followed exponential functions. Residuals of flutter phases could be detected up to *T*_a_ = 30.8 °C. Data at 40.6 °C were scarce and precarious and excluded from the fitting curves. Mean values are shown with SD. Number of evaluated respiration cycles shown beside the cycle data points. See Table 1 for functions and statistics

With rising ambient temperature, the duration of gas exchange cycles and its phases followed an exponential decline (Fig. 4; Table 1). At *T*_a_ = 2.9 °C a gas exchange cycle lasted 116.85 ± 40.13 min on average, decreasing to 32.3 ± 9.81 min at *T*_a_ = 10.3 °C and 1.23 ± 0.53 min at *T*_a_ = 26.4 °C. The high standard deviation in data points at low experimental temperatures (<11 °C) resulted from inter- and intraindividual differences in gas exchange patterns. Closed and flutter phases followed the course of cycle duration closely. Closed phases were detectable up to *T*_a_ = 26.4 °C, and flutter phases up to *T*_a_ = 31.0 °C. In relation to the other phase types, data progression of open phase duration did not drop so steeply. However, open phase durations changed substantially from 10.87 ± 3.91 min at *T*_a_ = 2.9 °C to 5.23 ± 3.16 °C at *T*_a_ = 10.3 °C and 0.94 ± 0.37 min at *T*_a_ = 26.4 °C. Between 10 and 15 °C a peculiar “step” occurred in the respiratory traits. The progression of cycle duration changed sharply. The calculated curves fitted better if the data above and below this step were treated separately (Fig. 4). At the highest *T*_a_ of 40.3 °C it was almost impossible to distinguish single gas exchange peaks. Therefore, to avoid overestimation of cycle durations, those data were excluded from the best-fit equations.

**Table 1:** Equation parameters of exponential fit curves for gas exchange cycle and cycle phase duration

$${\text{duration }}\left( { \hbox{min} } \right) \, { = }\,y_{0} + A \times e^{{R_{0} \times T_{a} \, (^\circ {\text{C}})}}$$						
		*y*_0_	*A*	*R*_0_	*R* ^2^	*N*
cycle	lo	924.15222	12445.61199	−0.23965	0.70787	11
	hi	20.20735	5868.07068	−0.17376	0.36181	24
flutter	lo	−25.09957	5064.91551	−0.16371	0.30697	10
	hi	11.6347	218.77498	−0.11277	0.19853	14
open	lo	334.34833	1952.53183	−0.60802	0.28893	11
	hi	32.10397	1082.57681	−0.15595	0.63559	24
closed	lo	82.33714	12221.45362	−0.30067	0.54748	11
	hi	−9268.40355	9714.01021	−0.00163	0.07823	12

lo = temperatures 2.4 to 10.3 °C; hi = temperatures 15.4 to 35.6 °C; *P* < 0.05 for all fitting equations; *N* = number of individuals showing the gas exchange patterns evaluated

### CO_2_ emission per gas exchange cycle and resting metabolic rate

**Fig. 5:**
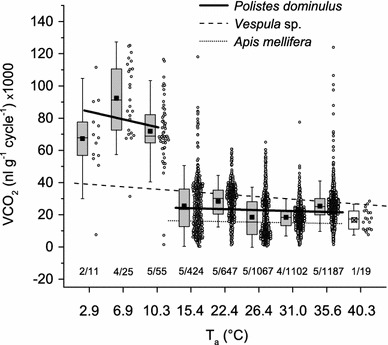
CO_2_ release per gas exchange cycle in resting *Polistes dominulus* at tested ambient temperatures (*T*_a_). The *circles* show single measurements from all individuals (overlapping values shifted horizontally). The *Boxplots* display Q1, Q2, Q3 and mean values (*black squares*). Whiskers indicate 1.5 interquartile range (IQR, def. Tukey). *Numbers* give the number of tested individuals/cycles. Number of measurements varied due to respiratory rates at different *T*_a_s. Regression lines were calculated from mean values of the individuals. The course of data showed a pronounced step between 10.3 and 15.4 °C, therefore the regression lines were calculated separately. At *T*_a_ = 40.3 °C only a few cycles from one individual were evaluable, therefore data were excluded from calculation. *P. dominulus* differs significantly (*P* < 0.0001, ANOVA) from *Vespula* sp. (dashed line, data from Käfer et al. 2012) in intercept (F-quotient = 566.04) and slope (F-quotient = 205.46) in the higher temperatures. In the lower temperatures, *P. dominulus* differs significantly from *Vespula* sp. in intercept (F-quotient = 241.86, *P* < 0.0001), but not in slope (F-quotient = 0.2, *P* > 0.5). *Polistes* differs also significantly (*P* < 0.0001, ANOVA) from *Apis mellifera* (*dotted line*, data from Kovac et al. 2007; intercept: F-quotient = 940.51; slope: F-quotient = 15.32)

**Fig. 6:**
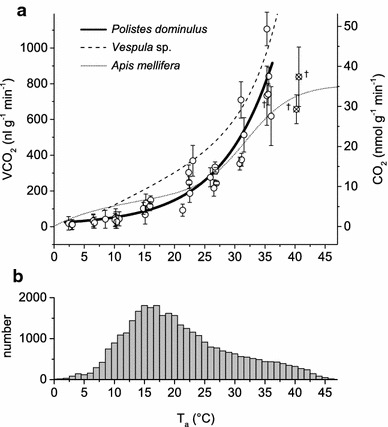
**a** Resting metabolic rate (CO_2_ release) of *Polistes dominulus* dependent on ambient temperature (*T*_a_). Each *data point* represents the mean value of one individual. Data follow an exponential course (*solid line*, ${\text{VCO}}_{ 2} \left( {{\text{nl g}}^{ - 1} {\text{min}}^{ - 1} } \right)\, =\, y_{0} + A \times e^{{R_{0} \times T_{\text{a}} \, \left( {^\circ C} \right)}}$). The *data points* at the highest temperature were marked because of possible impairment (adverse long-time temperature effects) of the wasps and excluded from the fitting equation. Individuals marked with *dagger* did not survive the experiment. *N* = 2–5 individuals per temperature range with *n* = 5–78 measurements per individual. Metabolism data from *Apis mellifera* (*dotted*, from Kovac et al. 2007) and *Vespula* sp. (*dashed*, from Käfer et al. 2012) are quoted for comparison. **b** Number of ambient temperature (*T*_a_) measurements in intervals of 15 min throughout the breeding season (April to October) at the surface of one nest each in 2012 and 2013

In resting *Polistes dominulus* the amount of CO_2_ released per gas exchange cycle increased abruptly below 15 °C (Fig. 5). Therefore, the data from the lower temperature categories (*T*_a_ = 2.9–10.3 °C) and those from the higher temperatures (*T*_a_ = 15.4–35.6 °C) were treated separately. Individual measurements showed a great variance. Dependence of mean CO_2_ release per gas exchange cycle on temperature showed no significance in the lower temperature range, but was significant in the higher temperature range. Parameters of linear regression fits of ${\text{VCO}}_{ 2} \left( {{\text{nl g}}^{ - 1} {\text{cycle}}^{ - 1} } \right) = a + b \, \times \, T_{\text{a}} \left( {^\circ {\text{C}}} \right)$ were: *a* = 88.48445 and *b* = −1.31815 for the lower temperatures (*R*^2^ = 0.0094, *P* = 0.17785, *n* = 90, 11 animals), and *a* = 26.13804 and *b* = −0.13121 for the higher temperatures (*R*^2^ = 0.00429, *P* < 0.0001, *n* = 4423, 24 animals). If estimated from the fitting lines, CO_2_ emission per cycle decreased from 84,667 nl g^−1^ min^−1^ at *T*_a_ = 2.9 °C to 74,776 nl g^−1^ min^−1^ at *T*_a_ = 10.4 °C in the lower temperature range, and from 24,118 nl g^−1^ min^−1^ at *T*_a_ = 15.4 °C to 21,467 nl g^−1^ min^−1^ at *T*_a_ = 35.6 °C in the higher range. *Polistes* exhaled significantly more CO_2_ per cycle at lower *T*_a_ (2.9–10.3 °C) than at higher *T*_a_ (15.4–35.6 °C); the difference in mean CO_2_ emission was 54,410 nl g^−1^ cycle^−1^ or 3.4 fold (*P* ≤ 0.0001, *U* test). The mass-specific resting metabolism was calculated from the individuals’ average carbon dioxide release rate (VCO_2_) and their body weight was determined prior to the experiment. Data progression followed an exponential course with increasing experimental ambient temperature, from 18.97 nl g^−1^ min^−1^ at *T*_a_ = 2.9 °C to 90.21 nl g^−1^ min^−1^ at *T*_a_ = 15 °C, 267.39 nl g^−1^ min^−1^ at *T*_a_ = 25 °C and 759.23 nl g^−1^ min^−1^ at *T*_a_ = 35 °C (Fig. 6; Table S3). The following equation fitted the data best:

$${\text{VCO}}_{ 2} \left( {{\text{nl g}}^{ - 1} {\text{min}}^{ - 1} } \right)\, =\, y_{0} + A \times e^{{R_{0} \times T_{\text{a}} \, \left( {^\circ {\rm C}} \right)}}$$

Parameters are *y*_0_ = 6.78686, *A* = 14.30461, *R*_0_ = 0.11472 (*R*^2^ = 0.89819; *P* < 0.0001; 28 individuals; *n* = 990 10 min intervals, 5–78 per individual; range of validity is 2.9–35.6 °C).

**Fig. 7:**
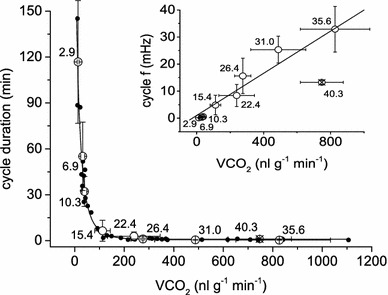
Gas exchange cycle duration (min) as a function of CO_2_ release (VCO_2_).* Large data point*s are mean values with SD.* Numbers beside the data points* indicate the tested ambient temperature in °C.* Small data points* indicate mean values for the single individuals. The following equation calculated from these points fitted the data best: ${\text{cycle duration }}\left( { \hbox{min} } \right) \,{ = }\,y_{0} + A_{1} \times e ^{-{\left( {{\text{VCO}}_{ 2} - x_{0} } \right)/t_{1} }} + A_{2} \times e ^{-{\left( {{\text{VCO}}_{ 2} - x_{0} } \right)/t_{2} }} + A_{3} \times e ^{-{\left( {{\text{VCO}}_{ 2} - x_{0} } \right)/t_{3} }}$ with *y*_0_ = 54.6712; *x*_0_ = 13.63852; *A*_1_ = 2770.14182; *t*_1_ = 15.00212; *A*_2_ = 1067.54685; *t*_2_ = 15.01563; *A*_3_ = 2379.78549; *t*_3_ = 44.56732; VCO_2_ in nl g^−1^ min^−1^. The *insert* shows respiration cycle frequency (*f*) as a function of VCO_2_.* Numbers beside data points* indicate the tested ambient temperature in °C. Linear fit: ${\text{cycle frequency }}\left( {\text{mHz}} \right) \, { = } \, a + b \times {\text{VCO}}_{ 2} \left( {{\text{nl }} g^{ - 1} {\text{min}}^{ - 1} } \right)$; *a* = 1.00166, *b* = 0.03896 (*R* ^2^ = 0.83011, *N* = 29, *P* < 0.0001); Data at 40.3 °C were excluded from the fitting calculations

Cycle duration as a function of CO_2_ release (VCO_2_) calculated from the mean values of individuals followed an exponential course (Fig. 7). Gas exchange cycle frequency (*f*) as an inverse of cycle duration was linearly dependent on VCO_2_ (Fig. 7, insert). Data at 40.3 °C were excluded from the calculation of fitting lines because no wasp survived the experiment (see Sect. “Mortality at high *T*_a_”).

### Mortality at high *T*_a_

Up to *T*_a_ = 35.6 °C all individuals but one survived the experimental temperatures they were exposed to. At the highest experimental temperature of *T*_a_ = 40.3 °C, none of the five tested wasps survived the experiments. Three individuals did not show resting behaviour at all. 11.0, 6.9 and 3.1 h after the experiment had started they showed spasms and died. Their gas exchange patterns became erratic at this time. As they were never at rest during the entire experiment, these individuals are not represented in this analysis. Only two of the five tested individuals showed short periods of rest briefly before gas exchange became erratic and controlled movement stopped, which occurred 6.1 and 6.6 h after the experiment had started.

### Lower thermal limit

All nine individuals were at rest soon after insertion into the measurement chambers. Even after being forcefully shaken, most of them showed sparse movement, mainly to regain or increase hold in their previous position. With lower temperatures, voluntary movements as well as direct reactions to shaking became slower and less frequent. Locomotion ceased between *T*_a_ = 5 and 2.5 °C. At lower temperatures individuals fell on their back or side and remained in this position, trying to right themselves—mostly unsuccessfully—with leg movements, sometimes combined with slow twisting of the abdomen. Coordinated reactive movements ceased at *T*_a_ = −3.0 ± 0.9 °C. All individuals survived −5 °C for 5 min and showed normal behavioural reactions after being warmed up at the end of the experiment.

## Discussion

We provide the first comprehensive investigation of the resting (standard) metabolism (Willmer et al. 2004) of *Polistes dominulus* over most of the temperature range this species could be exposed to during a breeding season in Central Europe. This gives insights into the species’ energetic requirements in its highly variable thermal environment.

### Weight and fuel

The standard metabolism of organisms is usually measured in the post-absorptive state to exclude a rise of metabolic rate during digestion (Frappell and Butler 2004, Hulbert and Else 2004, McNab 1997), i.e. tested animals are starved for some time before and during the experiment. This does not always lead to valid results (see DeVries et al. 2015), especially in species with small energy reserves (McDevitt and Andrews 1995, Rezende et al. 2011, Santos et al. 2011). In endothermic insects like paper wasps, vespine wasps or honeybees, long-term experiments on fasting individuals may lead to impairment and even death. This is especially important at higher experimental temperatures (~40 °C), where our wasps were agitated and displayed only few and short resting periods, if any.

A mean weight gain of 6 mg in surviving individuals showed that the animals did feed on the sucrose solution provided. Individuals not surviving the experiments did not feed for extended periods despite showing high body activity (see also Overgaard et al. 2012). Their weight probably also diminished from water loss because of excessive evaporative cooling (see also Weiner et al. 2010), respiratory water loss and uncontrolled evaporation after death. The provision of a food source to fuel the tested individuals and to support their water balance was therefore indispensable.

### Body temperature and activity

There is a close relationship between endothermic activity and metabolic rate (Bartholomew and Casey 1978, Bartholomew and Lighton 1986, Chappell and Morgan 1987, Heinrich 1974, 1993). Therefore, in heterothermic insects, measuring the body temperature is essential for the evaluation of the standard metabolic rate. At the lower experimental temperatures (*T*_a_ ~ 5–15 °C), most resting *P. dominulus* were weakly endothermic (*T*_th_ − *T*_ab_ > 1.0 °C, Fig. 2), similar to *Vespula* sp. (Käfer et al. 2012). Results of Weiner et al. (2010) confirm this observation as they found that the body of five out of six inactive *P. dominulus* being kept at 8 °C for 10 min was slightly warmer than the environment. We suggest that the weak endothermic behaviour could serve to ward off cold torpor and shortening of the individual’s reaction time to exterior stimuli.

### Respiratory patterns and temperature

At temperatures ≤10 °C the wasps were at rest for the entire experiment. One might be tempted to interpret this inactivity as chill coma, following the original definition regarding body activity (Hazell and Bale 2011, MacMillan and Sinclair 2011, Rossbach 1872). The change in gas exchange rate and gas exchange pattern between 15 and 10 °C (Figs. 4, 6) might support this interpretation. However, it has to be noted that the wasps were able to control gas exchange well below 10 °C (see Fig. 3a). At the lowest experimental temperatures, CO_2_ emission was low due to a massively reduced metabolism (Fig. 6). The gas exchange pattern consisted of closed and flutter phases, and open phases with an initial larger peak and subsequent, merging smaller ones (Fig. 3a). This pattern within the open phase is also known from *Sisyphus fasciculatus* dung beetles at 20 °C (Duncan and Byrne 2000). In *Vespula* sp. we had observed such patterns at low ambient temperatures (below 10 °C; Käfer 2013, Käfer et al. 2013). This is different from *Apis mellifera*. Honeybees as an original tropical species display continuous CO_2_ emission below 10–11 °C (Lighton and Lovegrove 1990, Kovac et al. 2007) which indicates an ongoing failure of spiracle control. They fall into chill coma already at ~10 °C (Free and Spencer-Booth 1960, Goller and Esch 1990, Hosler et al. 2000). However, *Polistes* reacted immediately, though sluggishly, to external stimuli (e.g. opening of the measurement chamber at the end of the experiment) after 11 h of experiment, even at the lowest experimental temperatures. In the lower temperature limit experiments, some of them even crawled slowly through the experimental vials at temperatures of 5–2.5 °C.

A surprising finding of the present investigation was that the CO_2_ release pattern of *P. dominulus* changed massively between 15 and 10 °C. This appeared as a pronounced break in the data progression regarding cycle duration (Fig. 4). We suggest that this is an adaptation of respiratory control to specific requirements at low temperatures, which is supported by the fact that CO_2_ emission per cycle increased abruptly (~2.8-fold, Fig. 5) between 15 and 10 °C whilst the correlation of metabolic rate (VCO_2_) with gas exchange frequency (*f*) showed no such pronounced step in the data course (Fig. 7). Would the change in respiratory patterns just have been the result of a drop in metabolic rate, this should not have occurred. Results suggest that either the trigger threshold for CO_2_ release has increased or the buffer capacity of the haemolymph at low temperature changed (see Förster and Hetz 2010, Levy and Schneiderman 1966). A comparison of CO_2_ emission per gas exchange cycle data revealed that *P. dominulus* exhaled a smaller amount of CO_2_ than *Vespula* sp. but more than *A. mellifera* above the respiratory break (≥15 °C; *P* < 0.0001, Fig. 5). Below this break (≤10 °C), where only *Vespula* data are available, *P. dominulus* exhaled a significantly higher amount per cycle (*P* < 0.0001, Fig. 5). These findings comply with the generally lower metabolic rate of *Polistes*, especially at low temperatures. However, the exact physiological mechanisms acting during this respiratory break (transition) remain to be elucidated.

### Metabolism, ambient temperature and lifestyle

Resting metabolic rate (CO_2_ emission per time) of *Polistes* is below that of *Vespula* sp.—a “high energy turnover” hymenopteran—in the entire tested temperature range (Fig. 6) despite similar size and body mass. This seems to support the aerobic capacity hypothesis which states that the lower active metabolic capacity of *Polistes* (Weiner et al. 2009, 2012) should be reflected in a lower standard metabolic rate (Bennett and Ruben 1979, Hayes and Garland 1995). However, this conclusion seems not to be valid in comparison to honeybees. The resting metabolism of *Polistes* is below that of the honeybees at low temperatures (<25 °C), but higher in the upper temperature range (>25 °C).

A low standard metabolic rate (SMR) has been suggested to be an adaptation to arid conditions (Lighton and Bartholomew 1988). In a warm environment this is thought to reduce energetic needs. The original provenance of *P. dominulus* is around the Mediterranean Sea, in Southern Europe and Northern Africa with hot, dry summers. Therefore, the low resting metabolism of *P. dominulus* matches this theory. In addition, a low SMR is probably beneficial in the energy-extensive lifestyle of the European paper wasp (see Reinhold 1999). The animals show long periods of rest and are hardly endothermic on the nest (our own observations; compare Höcherl and Tautz 2015). They do not actively heat to achieve a constant nest temperature for accelerated brood development even at lower ambient temperatures and protect the nest only from overheating when it gets hot. Heat from a high SMR would not contribute to a faster brood development like in the isolated nests of vespine wasps or honeybees, but would immediately be lost to the environment. A low SMR reduces this heat loss, and in addition saves energy which otherwise would be necessary for additional foraging flights. Our analysis of the thermal regime during a breeding season shows that resting *Polistes* (in contrast to honeybees) are mostly in the lower range of their SMR curve at the nest: 50 % of cumulative ambient temperature values were below 18.9 °C and 75 % below 25.8 °C (Fig. 6b). Below ~20 °C we observed a generally reduced activity. If less energy is required at rest, but also for thermoregulation and foraging, the food requirement for the adult individuals is lower and more food is available for brood rearing. The amount of brood can therefore be increased and brood development can be accelerated (Weiner et al. 2012).

Global climate change with the associated rise in average annual temperature should also influence the survival strategies and abundance of *P. dominulus* considerably. The spread of their habitat to colder areas such as northern Europe could, in addition to other important factors like warmer temperatures during overwintering, have been promoted by a shift in the mean ambient temperatures to the optimum range of activity and brood development of *Polistes dominulus*, and so could have enabled the population to colonise formerly unsuitable habitats.

## Electronic supplementary material

Supplementary material 1 (PDF 103 kb)
