# Supplementary material for: Respiration and metabolism of the resting European paper wasp (Polistes dominulus)
Source: J Comp Physiol B. 2015 Jul 2;185(6):647–58. doi: 10.1007/s00360-015-0915-7 (PMC4506450; doi:10.1007/s00360-015-0915-7)
Supplement: Supplementary file 1 — Supplementary material 1 (PDF 103 kb) [file 360_2015_915_MOESM1_ESM.pdf]

Table S1 Temperature categories and individual (NXX) experimental ambient temperature

	**2.9 °C**
N23	2.4
N25	2.9
N26	3.0

	**6.9 °C**
N19	6.5
N20	6.6
N21	6.7
N12-13	8.5

	**10.3 °C**
N14	10.2
N15	10.2
N16	10.8
N17*	10.1
N18	10.4

	**15.4 °C**
N08	15.1
N09	15.3
N10	14.9
N14-13	16.0
N16-13	16.0

	**22.4 °C**
N01[2]	22.5
N02[2]	22.3
N03[2]	22.4
N04-13	23.0
N30	21.3

	**26.4 °C**
N01[2]	26.9
N02[2]	26.7
N03[2]	26.8
N04	26.5
N05-13	25.9

	**31.0 °C**
N05[2]	31.0
N06[2]	31.1
N06-13	30.8
N28	31.5

	**35.6 °C**
N05[2]	35.3
N06[2]	35.3
N07[†]	35.5
N11-13	35.6
N29	36.0

	**40.3 °C**
N11[†]	40.3
N12** [†]	40.4
N13[†]	40.6
N27** [†]	40.2

N*XX* – individuals from 2011; N*XX*-13 – individuals from 2013
[2] individuals were tested at two temperatures
* CO_2 peaks not analyzed; ** no rest; [†] dead at the end of experiment

Figure S2 Body mass change in *Polistes* individuals and relative humidity (rH) at experimental ambient temperatures (T_a).

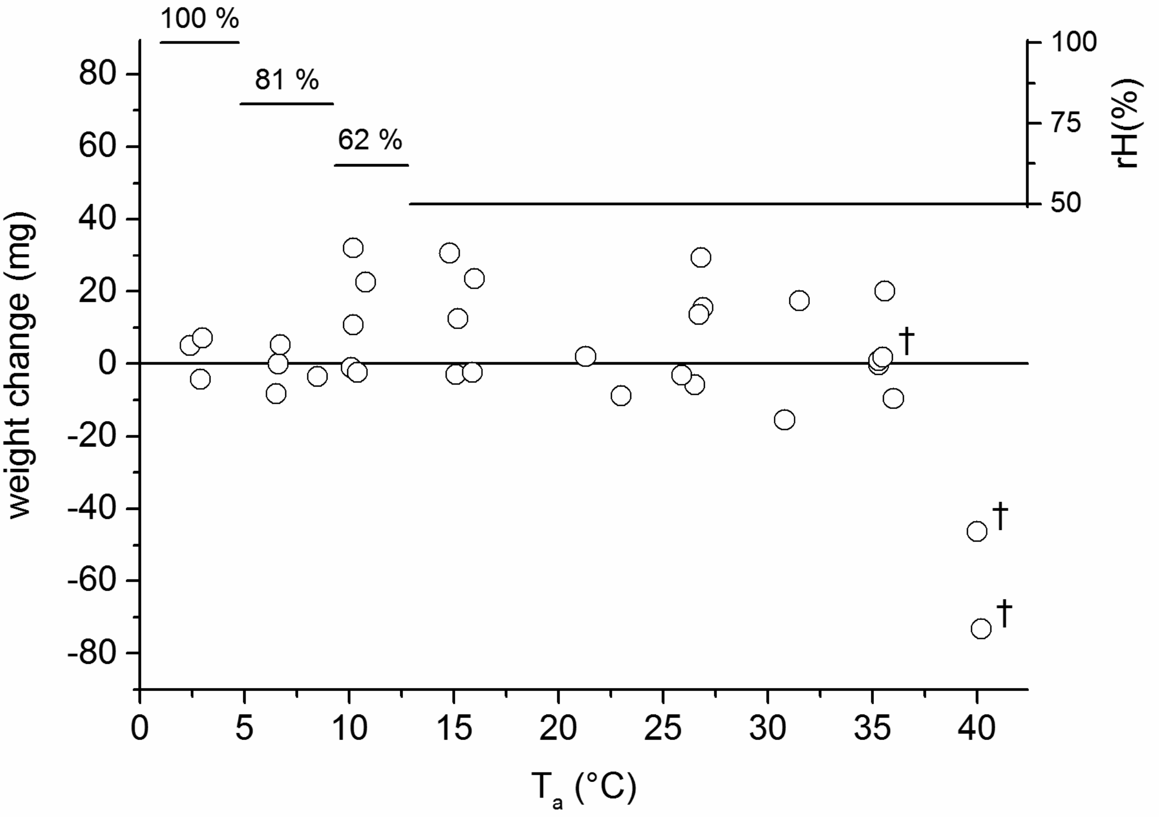

† dead at the end of experiment

Table S3 Mean CO_2 emission (VCO_2) of *Polistes dominulus* at experimental ambient temperatures (T_a, categories)

T_a (°C)	VCO_2 (nl g^{-1} min^{-1})	SD	n (averaged 10 min intervals)	N (individuals)	weight (g)	SD
2.9	11.88	28.14	131	3	0.0963	0.0081
6.9	29.86	37.18	168	4	0.0948	0.0932
10.3	39.91	43.38	181	5	0.0861	0.0136
15.4	115.01	47.97	153	5	0.0892	0.0154
22.4	256.42	118.05	114	5	0.0837	0.0087
26.4	276.56	60.97	112	5	0.0810	0.0160
31.0	435.07	133.55	70	4	0.0965	0.0058
35.6	773.47	152.62	51	5	0.0802	0.0202
40.3	772.61	193.44	19	2	0.1196	0.0175

Number of evaluated 10 minute intervals (n) and individuals (N)
Mean weight data (g) per temperature category

Figure & Table S4 Respiration cycle frequency (f) and cycle phases as a function of ambient temperature (T_a) displayed in logarithmic scale. Data points are mean values at tested T_a - category. The "step" in the data course results from changes in respiration patterns between 10 and 15 °C. Regression lines were calculated below and above the "step". Number of evaluated respiration cycles shown beside the cycle data points. At T_a = 40.6 °C respiration data (marked) were scarce and precarious and excluded from the fitting line.

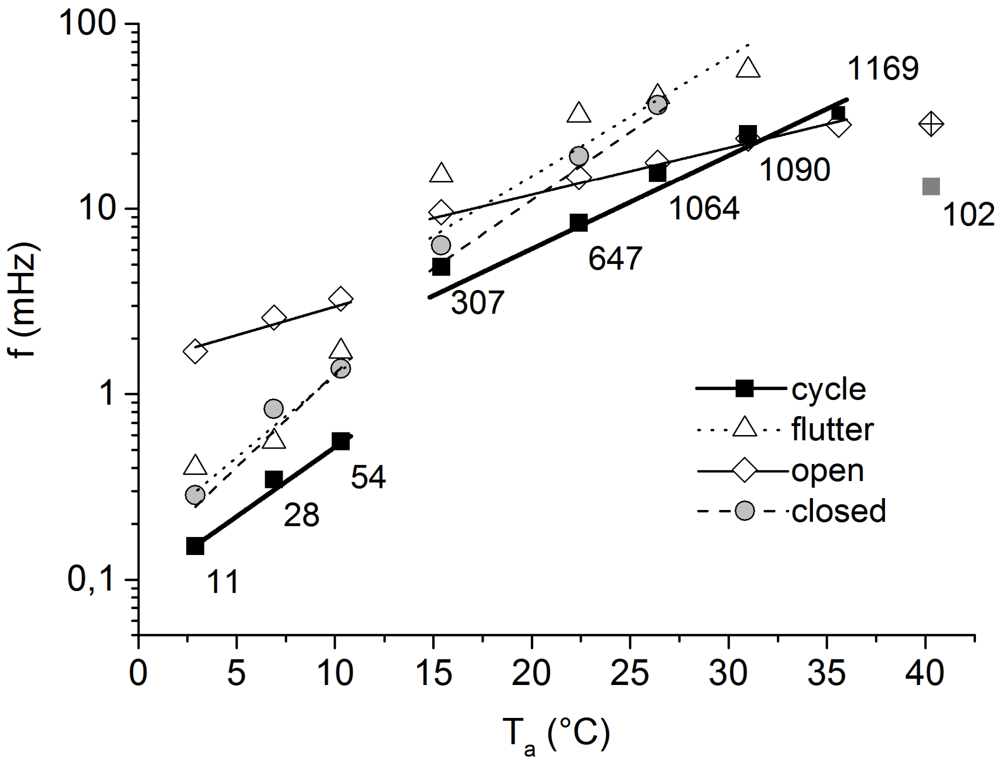

f (mHz) = a + b * T_a *(°C)*

		a	b	R^2	N	slopes	
cycle	lo	-1.03392	0.07485	0.71543	11	] **	p < 0.002
	hi	-0.22376	0.05043	0.7137	24		F-Quotient = 9.52
flutter	lo	-0.78218	0.0884	0.38968	10	] **	p < 0.01
	hi	-0.1136	0.06451	0.54848	14		F-Quotient = 23.78
open	lo	0.16631	0.0307	0.16016	11	] ***	p < 0.0001
	hi	0.56882	0.02543	0.71125	24		F-Quotient = 15.85
closed	lo	-0.9126	0.10151	0.66732	11	] ***	p < 0.0001
	hi	-0.42375	0.07359	0.59261	12		F-Quotient = 15.83

Parameters and statistics of linear regressions. lo = temperatures of 2.4 to 10.3 °C; hi = temperatures of 15.4 to 35.6 °C; P < 0.05 for all fitting equations, except for "open lo" P = 0.12239. Slopes differ significantly between hi and lo temperatures in cycles and all cycle components. N = number of individuals showing the gas exchange patterns evaluated
